# Rheumatoid arthritis is associated with reduced adiposity but not with unfavorable major cardiovascular risk factor profiles and enhanced carotid atherosclerosis in black Africans from a developing population: a cross-sectional study

**DOI:** 10.1186/ar4276

**Published:** 2013-08-22

**Authors:** Patrick H Dessein, Angela J Woodiwiss, Gavin R Norton, Ahmed Solomon

**Affiliations:** 1 Cardiovascular Pathophysiology and Genomics Research Unit, School of Physiology, Faculty of Health Sciences, University of the Witwatersrand, 7 York Road, Parktown, Johannesburg 2193, South Africa; 2 Department of Rheumatology, Charlotte Maxeke Johannesburg Academic Hospital, Faculty of Health Sciences, University of the Witwatersrand, 7 York Road, Parktown, Johannesburg 2193, South Africa

## Abstract

**Introduction:**

Rheumatoid arthritis (RA) is characterized by inflamed joint-derived cytokine-mediated high-grade systemic inflammation that enhances cardiovascular metabolic risk and disease in developed populations. We investigated the potential impact of RA on cardiovascular risk factors including systemic inflammation and atherosclerosis, and their relationships in black Africans from a developing population.

**Methods:**

We evaluated demographic features, adiposity indices, major traditional cardiovascular risk factors, circulating C-reactive protein and interleukin-6 concentrations and ultrasound determined carotid intima-media thickness (cIMT) in 274 black Africans; 115 had established RA. Data were analyzed in confounder-adjusted mixed regression models.

**Results:**

The body mass index and waist-height ratio were lower in RA compared to non-RA subjects (29.2 (6.6) versus 33.7 (8.0), *P *< 0.0001 and 0.58 (0.09) versus 0.62 (0.1), *P *= 0.0003, respectively). Dyslipidemia was less prevalent in patients with RA (odds ratio (OR) (95% confidence interval (CI) = 0.54 (0.30 to1.00)); this disparity was no longer significant after further adjustment for reduced adiposity and chloroquine use. RA was also not associated with hypertension, current smoking and diabetes. The number of major traditional risk factors did not differ by RA status (1.1 (0.8) versus 1.2 (0.9), *P *= 0.7). Circulating C-reactive protein concentrations were similar and serum interleukin-6 concentrations reduced in RA (7.2 (3.1) versus 6.7 (3.1) mg/l, *P *= 0.7 and 3.9 (1.9) versus 6.3 (1.9) pg/ml, *P *< 0.0001, respectively). The cIMT was 0.700 (0.085) and 0.701 (0.111) mm in RA and non-RA subjects, respectively (*P *= 0.7). RA disease activity and severity parameters were consistently unrelated to systemic inflammation, despite the presence of clinically active disease in 82.6% of patients. In all participants, adiposity indices, smoking and converting angiotensin inhibitor non-use were associated with increased systemic inflammation, which related to more atherogenic lipid profiles, and circulating low density lipoprotein concentrations were associated with cIMT (partial R = 0.153, *P *= 0.032); RA did not impact on these relationships (interaction *P *≥0.1).

**Conclusions:**

Among black Africans, patients with established RA experience reduced overall and abdominal adiposity but no enhanced major traditional risk factor and atherosclerosis burden. This study further suggests that an absent interleukin-6 release by inflamed RA joints into the circulation may account for this unaltered cardiovascular disease risk.

## Introduction

Rheumatoid arthritis (RA) is a chronic inflammatory and potentially destructive joint disorder that is complicated by enhanced atherosclerosis and incident cardiovascular event rates similar to diabetes [1-6], as well as cardiovascular mortality [4,7].

In the general population, the bulk of cardiovascular disease (CVD) is attributable to the traditional risk factors of hypertension, dyslipidemia, smoking and diabetes [8,9]. In addition, cytokine-mediated systemic inflammation as generally estimated by circulating C-reactive protein concentrations (CRP), contributes to atherogenesis [10-19]. Systemic inflammation originates mostly in excess adiposity and smoking, and mediates CVD through adverse effects on metabolic cardiovascular risk factors as well as more direct effects at the endothelial level [10-19].

RA is characterized by circulating CRP concentrations that are typically increased several fold even when the disease appears clinically controlled [20,21]. Although, in RA, adiposity and smoking associate independently with serum CRP concentrations [22], the inflammation extent is mostly accounted for by circulating joint derived cytokines, particularly interleukin-6 [20-22].

Several genetic polymorphisms were recently found to be associated with CVD in RA [23-27]. Amongst the modifiable cardiovascular risk factors, it is the chronic high-grade systemic inflammatory state that is currently considered to best explain the excess CVD in RA [20,21,28-31]. Congruent with this paradigm, systemic inflammation is associated with metabolic risk factors, including insulin resistance and decreased high density lipoprotein (HDL) cholesterol concentrations in RA [20,21,32,33]. A recent meta-analysis of traditional cardiovascular risk factors in RA indeed confirmed the presence of lower HDL cholesterol concentrations and an enhanced diabetes frequency [34]. Importantly in the present context also, high-grade inflammation in RA is complicated by a reduced lean mass and particularly muscle mass together with increased body fat accumulation, a condition most often termed rheumatoid cachexia [35,36]. Adiposity associates with metabolic risk factors in RA [36-38]. With regard to more recently identified cardiovascular risk factor pathways, circulating interleukin-6 concentrations are independently associated with endothelial activation [21] that decreases upon suppression of interleukin-6 production in RA [39].

Besides systemic inflammation in RA, antirheumatic agents can modify cardiovascular risk factors. Although short term glucocorticoid therapy in patients with markedly active RA can enhance insulin sensitivity [20], chronic use of this intervention associates with insulin resistance [40], atherosclerosis [41] and cardiovascular event rates [42]. Chloroquine therapy induces favorable lipid profiles [43,44] and lowered diabetes risk [45] and leflunomide use hypertension [46] and dyslipidemia [47].

As applies to the general population, available data on atherogenesis in RA were generally obtained in subjects that belong to developed populations, whereas 80% of CVD now occurs in low income or developing countries [38,48-52]. In this regard, we recently documented consistent disparities in individual cardiovascular risk factor profiles including more marked overall adiposity, an increased prevalence of hypertension and less frequent alcohol consumption in patients with RA from developing groups of black African descent compared to their white counterparts from a developed population, as well as risk factor-atherosclerosis relationships amongst both groups [38,51,52]. Non-RA black African subjects experience not only a very large prevalence of obesity [53] and hypertension [54,55], but also a strikingly large systemic inflammation burden [56-58], whereas serum C-reactive protein concentrations are not increased in black Africans compared to white patients with RA [52]. Further, cumulative inflammation as estimated by the number of deformed joints, is independently associated with decreased overall and abdominal obesity in African black but not white women with RA [38]. In the present study, we examined the potential impact of RA on cardiovascular risk factors, including systemic inflammation, carotid atherosclerosis and their relationships amongst 274 African blacks.

## Materials and methods

### Study participants

The present investigation was conducted according to the principles outlined in the Helsinki declaration. The Committee for Research on Human Subjects of the University of Witwatersrand approved the protocols applied in non-RA and RA subjects (approval numbers: M02-04-72 and renewed as M07-04-69 in non-RA subjects and M06-07-33 in RA subjects). Participants gave informed, written consent. The present study design has previously been described [38,50-52,56,59-61]. Briefly, 115 African black patients who met the 1988 American College of Rheumatology criteria for RA [62] were enrolled at the Charlotte Maxeke Johannesburg Academic Hospital. All invited patients agreed to participate. Only two RA subjects used prednisone and, hence, to avoid confounding of the data analysis by this intervention, the respective participants were excluded. All patients used disease modifying agents for rheumatic disease (DMARDs) at the time of the study. Age- and sex-matched non-RA subjects were participants in a population study on cardiovascular risk and disease that is also conducted in Johannesburg [56,59-61]. This investigation comprises randomly recruited nuclear families of black African descent with siblings older than 16 years. Of the 159 non-RA participants, 124 had CRP measurements and 92 carotid ultrasound evaluations; the other recorded variables did not differ in non-RA subjects with and without CRP and carotid ultrasound assessments. Data were missing in fewer than 5% of any of the other recorded cardiovascular risk factors in the study participants.

### Baseline characteristics

We recorded demographic features, life style factors comprising alcohol use (at least one unit per month) and exercise (at least once per month) that included time spent in walking, for example, to reach public transportation, and cardiovascular and non-steroidal anti-inflammatory drug (NSAID) use. Height, weight and waist and hip circumference were measured using standard approaches. Body mass index (BMI) cut-off points of <20, 20 to 24.9, 25 to 29.9 and >29.9 kg/m^2 ^were employed to identify underweight, normal weight, overweight and obese status, respectively. Abdominal obesity indices included waist circumference and waist-height ratio whereas fat distribution was estimated by the waist-hip ratio [38]. In patients with RA, we additionally recorded disease duration, the Clinical Disease Activity Index (CDAI) [63], the number of deformed joints (cumulative inflammation or disease severity), rheumatoid factor status and the use of traditional or synthetic DMARDs. Patients with RA had no access to biological DMARD therapy at the time of the study [38,50-52].

### Conventional cardiovascular risk factors

Hypertension was defined as an average systolic blood pressure ≥140 or/and diastolic blood pressure ≥90 mmHg or/and current use of antihypertensive medications. Uncontrolled and untreated hypertension were diagnosed in patients with a systolic blood pressure ≥140 or/and diastolic blood pressure ≥90 mmHg who used and did not use antihypertensive agents, respectively. Standard laboratory blood tests of renal and liver function, hematological parameters, lipids and glucose were performed. Dyslipidemia was diagnosed when the atherogenic index, that is, the cholesterol-HDL cholesterol ratio was more than four and proatherogenic non-HDL cholesterol concentrations were calculated by subtracting HDL cholesterol from total cholesterol concentrations [38,44,50-52]. We documented smoking habits. Diabetes was identified as the use of glucose lowering agents or a fasting plasma glucose ≥7 mmol/l. The overall major conventional cardiovascular risk factor burden was estimated by the number and proportion of patients with one or more of such risk factors that comprised hypertension, dyslipidemia, current smoking status and diabetes, as well as the Framingham 10-year risk prediction scores for coronary heart disease (myocardial infarction or coronary death) [8] and general CVD (myocardial infarction, coronary death, coronary insufficiency, angina, ischemic stroke, hemorrhagic stroke, transient ischemic attack, peripheral vascular disease or heart failure) [9].

### Systemic inflammation

Serum CRP and interleukin-6 comprised the evaluated laboratory inflammatory markers in the present investigation. CRP concentrations were determined using immunoturbidimetric methods. In non-RA subjects, this was done on the AU analyzer (Olympus, Essex, UK), the lower detection limit was 0.05 mg/l and the inter- and intra-assay coefficients of variation were 1.3 and 0.4%, respectively; in RA patients it was performed on the DxC/LX analyzer (Beckman Coulter, Inc., Brea, CA, USA), the lower detection limit was 1 mg/l and the inter- and intra-assay were 2.5 and 5.0%, respectively. In the general population, a CRP concentration >1 mg/l reportedly predicts increased incident CVD [16,19]. Seventy-four blood samples from subjects who did not participate in the present study were tested on both the AU and DxL/LX systems and the Spearman correlation coefficient between the obtained CRP values was 0.994. Interleukin-6 concentrations were measured using a solid-phase sandwich enzyme-linked immunosorbant assay (ELISA) (Quantikine^®^HS, R&D Systems, Inc., Minneapolis, MN, USA). The lower detection limit ranged from 0.016 to 0.110 pg/ml and inter- and intra-assay coefficients of variation were 7.8 and 7.4%, respectively.

### Carotid artery atherosclerosis

Carotid artery intima-media thickness (cIMT) measurements were made using a linear array 7.5 MHz probe attached to a high resolution B-mode ultrasound machine (SonoCalc IMT, Sonosite, Inc., Bothell, WA, USA) in both RA and non-RA subjects, as recently described [38,51,52]. This equipment involves the application of a unique semi-automated border detection program that was previously documented to provide highly reproducible intra- and inter-rater results in other as well as our settings [38,51,52,61,64]. Carotid artery plaque is currently identified in our RA patients [38,51,52] but not in non-RA subjects and, hence, results on plaque are not shown in the present report.

### Data analysis

Dichotomous variables are expressed as proportions or percentages and continuous variables as mean (SD). Non-normally distributed characteristics were logarithmically transformed prior to statistical analysis and for these variables geometric means (SD) are given. The selection of confounders in multivariable regression models was both data driven and based on biological plausibility.

Disparities in demographic features between RA and non-RA subjects were compared using the Student's *t*-test and univariate logistic regression analysis as appropriate. Relationships of RA with baseline recorded characteristics, major cardiovascular risk factors, systemic inflammation and atherosclerosis were first investigated in multivariable logistic and continuous regression models as appropriate with consistent adjustment for demographic characteristics since age differed numerically by RA status. The associations between RA and cardiovascular risk factors, systemic inflammation and atherosclerosis status were then re-assessed in models that included additional potential confounders or/and mediators.

In all participants, the relation of circulating CRP and interleukin-6 concentrations with potential determinants of systemic inflammation and metabolic cardiovascular risk factors were investigated in demographic characteristic as well as in multiple confounder adjusted models. The impact of RA on these relationships was determined by employing interaction terms [38,51,52]. Finally, in patients with RA, the associations of clinical disease activity and severity variables with systemic inflammation were investigated in demographic and multiple confounder adjusted models.

Statistical computations were made using the GB Stat™ program (Dynamic Microsystems, Inc., Silverspring, MD, USA).

## Results

### Baseline characteristics in African black subjects with and without RA

Table 1 gives the baseline characteristics in the study participants. The proportion of women was numerically larger by 3.4% amongst those with compared to those without RA. Alcohol was consumed only by non-RA subjects (17.6%), who also exercised more than their RA counterparts. The BMI was substantially lower in RA compared to non-RA subjects (difference in mean = 4.5 kg/m^2^). In age and sex adjusted analysis, black Africans with RA had more frequently a normal BMI (odds ratio (OR) (95% confidence interval (CI)) = 3.42 (1.69 to 6.95)) and were less often obese (OR (95% CI) = 0.35 (0.21 to 0.59)) than those without RA. Abdominal obesity measures (waist and waist-height ratio) were larger in non-RA subjects whereas RA was not associated with an altered fat distribution (waist-hip ratio). Hypertension was treated in 72.1% and 61.5% (OR (95% CI) = 1.76 (0.93 to 3.33)) of RA and non-RA cases, respectively. Antihypertensive agents were more frequently employed (OR (95% CI) = 1.87 (1.12 to 3.11)) and the mean number of antihypertensives prescribed was twice as large in subjects with compared to those without RA; this was mostly due to a more regular use of angiotensin converting enzyme inhibitors (OR (95% CI) = 10.00 (4.80 to 20.85)) and calcium channel blockers (OR (95% CI) = 4.01 (1.66 to 9.69)). Amongst treated hypertensive participants, 58.5% of non-RA subjects compared to 67.7% of RA patients had uncontrolled hypertension (systolic blood pressure ≥140 or/and diastolic blood pressure ≥90 mm Hg) (OR (95% CI) = 1.49 (0.71 to 3.14)).

**Table 1:** Baseline characteristics in African black subjects with and without rheumatoid arthritis

	Rheumatoid arthritis		
		
Characteristic	Present (*n *= 115)	Absent (*n *= 159)	*P*-value^a^
Demographics			
Age, years	55.7 (10.3)	56.5 (10.9)	0.5
Female (%)	89.6	86.2	0.45
Lifestyle factors			
Alcohol use (%)	0	17.6	**-**
Units per week, number^b^	0	0.27 (1.08)	**-**
Exercise (%)	41.7	42.8	1.0
Hours per week, number^b^	**0.1 (1.0)**	**1.7 (2.4)**	**0.0004**
Anthropometric measures			
Body mass index	**29.2 (6.6)**	**33.7 (8.0)**	**<0.0001**
<20 kg/m^2 ^(%)	5.4	2.5	0.2
20 to 24.9 kg/m^2 ^(%)	**25.2**	**8.8**	**0.0004**
25 to 29.9 kg/m^2 ^(%)	25.2	23.3	0.8
>29.9 kg/m^2 ^(%)	**44.1**	**64.8**	**0.0001**
Waist circumference, cm	**93.1 (13.3)**	**97.5 (15.0)**	**0.01**
Waist/height	**0.58 (0.09)**	**0.62 (0.1)**	**0.0003**
Hip circumference, cm	**110 (18)**	**116 (15)**	**0.002**
Waist/hip^b ^	0.85 (1.14)	0.84 (1.13)	0.4
Cardiovascular drugs			
Antihypertensive agents			
Use (%)	**53.9**	**40.2**	**0.02**
Number	**1.0 (1.1)**	**0.5 (0.7)**	**<0.0001**
>1 agent (%)	**36.5**	**8.8**	**<0.0001**
Diuretic (%)	38.3	39.0	1.0
Angiotensin converting enzyme inhibitor (%)	**40.8**	**6.9**	**<0.0001**
Calcium channel blocker (%)	**16.5**	**5.0**	**0.002**
Beta blocker (%)	3.5	0	**-**
Angiotensin receptor blocker (%)	0.9	0	**-**
Glucose lowering agents			
Oral glucose lowering agent (%)	13.9	10.1	0.3
Insulin (%)	0.9	2.5	0.3
Statin (%)	19.1	0	**-**
Nonsteroidal antiinflammatory agent (%)	6.1	4.4	0.6
Rheumatoid arthritis characteristics			
Disease duration, years	12.5 (8.8)		
Clinical Disease Activity Index^b ^[58]	8.3 (2.6)		
<2.8 or remission (%)	17.4		
2.7 to 10 or mild disease activity (%)	29.6		
11 to 22 or moderate disease activity (%)	40.0		
>22 or high disease activity (%)	13.0		
Deformed joints, number^b^	6.2 (2.7)		
Rheumatoid factor positive (%)	76.5		
Disease modifying agents			
Methotrexate (%)	92.2		
Chloroquine (%)	80.9		
Sulphasalazine (%)	25.2		
Leflunomide (%)	20.0		
Azathioprine (%)	14.8		
Tetracyclin (%)	10.4		
Cyclophosphamide (%)	6.1		
Penicillamine (%)	2.6		

Results are expressed as mean (SD) or proportions/percentages. ^a^Except for associations between rheumatoid arthritis status and demographic characteristics, adjusted for age and gender; ^b^non-normally distributed variables for which geometric mean (SD) is given.

In patients with RA, the mean disease duration was 12.5 years, 76.5% tested rheumatoid factor positive, 17.4% experienced clinical remission and 53% moderate or high disease activity. Methotrexate and chloroquine comprised the most frequently prescribed DMARDs and the mean number of DMARDs used was 2.4.

### Conventional cardiovascular risk factor profiles in African black subjects with and without RA

Table 2 shows the conventional cardiovascular risk factor profiles in black Africans with and without RA. In age- and sex-adjusted analysis (Model 1 in Table 2), RA was associated with less frequent dyslipidemia (OR (95% CI) = 0.54 (0.30 to 1.00)) and consistently more favorable individual lipid parameters. Subjects with RA had a similar frequency of ever smoking but had discontinued smoking more often than their non-RA counterparts. Amongst smokers, the mean number (SD) of cigarettes smoked per day was low at 4.1 (2.3). The overall major conventional cardiovascular risk factor burden as estimated by the number of major risk factors, the presence of at least one major risk factor and the 10-year risks for coronary heart disease and cardiovascular disease were similar in subjects with and without RA.

**Table 2:** Conventional cardiovascular risk factors, systemic inflammation and atherosclerosis in African black subjects by RA status

	Rheumatoid arthritis		Adjusted models				
		
Characteristic	Present (*n *= 115)	Absent (*n *= 159)	Model 1^a^	Model 2^b^	Model 3^c^	Model 4^d^	Model 5^e^
**Categorical variables**			**OR (95% CI)**	**OR (95% CI)**	**OR (95% CI)**	**OR (95% CI)**	**OR (95% CI)**
Hypertension (%)	74.8	65.4	1.65 (0.95 to 2.96)	1.66 (0.94 to 2.96)	**2.03 (1.07 to 3.85)**	-	1.88 (0.97 to 3.67)
T chol/HDL chol >4	21.6	33.5	**0.54 (0.30 to 1.00)**	0.73 (0.40 to 1.33)	0.64 (0.31 to 1.30)	0.87 (0.31 to 3.07)	0.88 (0.28 to 2.79)
Smoking							
Ever (%)	16.5	14.5	1.37 (0.66 to 2.85)	-	-	-	** -**
Former (%)	13.0	6.9	**2.48 (1.03 to 5.99)**	-	-	-	** -**
Current (%)	3.5	7.6	0.46 (0.14 to 1.51)	-	-	-	** -**
Diabetes (%)	15.7	12.0	1.41 (0.69 to 2.85)	1.87 (0.86 to 4.06)	-	1.48 (0.37 to 5.99)	1.53 (0.38 to 6.28)
≥1 major RF (%)	75.7	78.1	0.87 (0.49 to 1.53)	0.86 (0.48 to 1.53)	0.66 (0.24 to 1.81)	1.23 (0.34 to 4.12)	0.88 (0.28 to 3.79)
							
**Continuous variables**			**Partial R *P***	**Partial R *P***	**Partial R *P***	**Partial R *P***	**Partial R *P***
Blood pressure values							
Systolic BP, mmHg	140 (25)	137 (22)	0.064 0.3	0.035 0.6	0.080 0.2	-	0.092 0.2
Diastolic BP, mmHg	86 (15)	87 (13)	-0.038 0.5	-0.031 0.6	-0.008 0.9	** -**	-0.017 0.8
Lipid values							
T chol, mmol/l	4.7 (0.9)	5.1 (1.2)	**-0.145 0.02**	-0.112 0.08	**-0.126 0.045**	-0.075 0.2	-0.072 0.3
HDL chol^f^, mmol/l	1.48 (1.33)	1.39 (1.32)	0.112 0.07	0.082 0.2	0.094 0.1	-0.001 1.0	0.026 0.7
T chol/HDL chol	3.2 (1.0)	3.7 (1.3)	**-0.180 0.003**	**-0.134 0.03**	**-0.155 0.01**	-0.041 0.5	-0.064 0.4
LDL chol, mmol/l	2.6 (0.8)	3.0 (1.0)	**-0.173 0.005**	**-0.133 0.04**	**-0.146 0.02**	-0.096 0.1	-0.120 0.09
LDL chol/HDL chol^f^	1.68 (1.55)	2.02 (1.59)	**-0.179 0.004**	**-0.134 0.03**	**-0.147 0.02**	-0.070 0.3	-0.110 0.1
Non HDL, mmol/l	3.1 (0.9)	3.6 (1.2)	**-0.190 0.002**	**-0.145 0.02**	**-0.165 0.009**	-0.081 0.2	-0.090 0.2
Trig^f^, mmol/l	1.08 (1.70)	1.20 (1.59)	-0.118 0.06	-0.093 0.14	-0.119 0.06	-0.058 0.4	-0.041 0.6
Trig/HDL chol^f^	0.73 (2.02)	0.87 (1.83)	**-0.139 0.02**	-0.110 0.08	**-0.135 0.03**	-0.046 0.5	-0.046 0.6
Smoking^f^, cigarettes/d	0.05 (0.36)	0.15 (0.68)	-0.096 0.1	-	-	-	-
Glucose ^f^, mmol/l	5.2 (1.4)	5.5 (1.4)	-0.117 0.06	-0.090 0.2	-	-0.095 0.1	-0.088 0.2
Major RF, number	1.1 (0.8)	1.2 (0.9)	-0.024 0.7	0.010 0.9	0.008 0.9	0.035 0.6	0.023 0.7
10 year risk for CHD ^f^	2.5 (3.0)	3.2 (3.2)	-0.103 0.1	-0.090 0.2	-0.057 0.4	-0.042 0.5	-0.012 0.9
10 year risk for CVD ^f^	9.3 (2.2)	10.4 (2.4)	-0.050 0.4	-0.012 0.9	0.035 0.6	0.001 1.0	-0.002 1.0
Systemic inflammation							
CRP, mg/l ^f^	7.2 (3.1)	6.7 (3.1)	0.024 0.7	0.099 0.1	-	-	** -**
Interleukin-6, pg/ml ^f^	3.9 (1.9)	6.3 (1.9)	**-0.344 <0.0001**	**-0.296 <0.0001**	-	-	** -**
Atherosclerosis							
cIMT, mm	0.700 (0.085)	0.701 (0.111)	0.025 0.7	0.062 0.4	0.076 0.3	0.013 0.9	0.036 0.7

Results are expressed as mean (SD) or proportions/percentages. Significant associations are shown in bold. ^a^Adjusted for age and sex; ^b^additionally adjusted for body mass index and waist to height ratio; ^c^additionally adjusted for leflunomide use; ^d^additionally adjusted for chloroquine use; ^e^additionally adjusted for serum C-reactive protein and interleukin-6 concentrations; the number of antihypertensive agents employed and use of statins and oral glucose lowering agents as well as insulin were additionally adjusted for in models that included blood pressure and lipid variables and glucose concentrations, respectively; ^f^non-normally distributed variables for which geometric mean (SD) is given. RA, rheumatoid arthritis; BP, blood pressure; CHD, coronary heart disease; cIMT, common carotid intima-media thickness; CRP, C-reactive protein; CVD, cardiovascular disease; HDL chol, high density lipoprotein cholesterol; trig, triglycerides; LDL chol, low density lipoprotein cholesterol; RF, risk factors; T chol, total cholesterol

Besides disparities in adiposity measures between RA and non-RA subjects (Table 1), other factors that could have confounded or mediated our findings in Model 1 (Table 2) included chloroquine [43-45] and leflunomide use [46,47]. Thus, amongst patients with RA and in age, sex, cardiovascular drug, obesity measure and lifestyle factor adjusted analysis, potentially relevant relationships (*P *< 0.2) were found between chloroquine and leflunomide use and the cholesterol-HDL cholesterol ratio (partial R = -0.153, *P *= 0.13 and partial R = 0.158, *P *= 0.12, respectively). Leflunomide use also potentially impacted on systolic blood pressure but this constituted an inverse relationship (partial R = -0.177, *P *= 0.08) thereby arguing against an adverse effect of leflunomide on blood pressure amongst black Africans with RA. None of the other RA characteristics (Table 1) were found to be potential confounders or mediators in the associations between RA and cardiovascular risk factors (*P *> 0.2).

The mediating or confounding effects of disparities of adiposity indices between RA and non-RA subjects, leflunomide and chloroquine use and systemic inflammation on the associations between RA and cardiovascular risk factors (Model 1 in Table 2) are shown in models 2 to 5. In Model 2, additional adjustment for anthropometric measures consistently attenuated the inverse associations between RA and lipid variables. In Model 3, further adjustment for leflunomide use strengthened the respective associations thereby confirming an adverse effect of leflunomide on serum lipid concentrations. In keeping with the above reported negative borderline relationship between leflunomide use and systolic blood pressure, the association between RA and hypertension was also strengthened and, in fact, significant. In Model 4, additional adjustment for chloroquine use resulted in a complete lack of association between RA and lipid parameters. In Model 5, further adjustment for serum CRP and interleukin-6 concentrations did not materially alter the results in Model 4. The same applied when lifestyle factors were adjusted for (data not shown).

In a separate model, the number of antihypertensives used (Table 1) remained higher in RA compared to non-RA subjects independent of demographic characteristics, life style factors, obesity measures, systemic inflammation and leflunomide use; amongst patients with RA, none of the disease characteristics were related to the number of antihypertensives used (data not shown).

### Systemic inflammation in African black subjects with and without RA

In age and sex adjusted analysis, circulating interleukin-6 concentrations were strongly associated with circulating CRP concentrations (partial R = 0.396, *P *< 0.0001) and the presence of RA did not impact on this relationship (interaction *P *> 0.1)

Table 2 further gives serum CRP and interleukin-6 concentrations in the study subjects. Serum CRP concentrations did not differ and the serum interleukin-6 concentrations were lower in RA compared to non-RA subjects (*P *= 0.7 and *P *< 0.0001, respectively, in age and sex adjusted analysis) (Model 1 in Table 2). Adjustment for obesity measures did not materially alter these results (Model 2 in Table 2). Also, further adjustment for alcohol use, cigarettes smoked per day and the use of angiotensin converting enzyme inhibitors that were additional potential confounders in the present context (see analyses below), did not materially alter the relationship between RA and systemic inflammation (data not shown).

The lack of impact of RA on CRP concentrations and reduced interleukin-6 concentrations in patients with RA is unexpected. Therefore, we further measured CRP and interleukin-6 concentrations in 122 African whites with RA that formed part of an investigation that was previously reported by us [52]. We used the same assay as in all subjects and the one employed in non-RA subjects in the present study upon quantifying CRP and interleukin-6 concentrations (see methods), respectively. In African white patients with RA, the geometric mean (SD) CRP and interleukin-6 concentrations were 4.3 (3.7) mg/l and 3.6 (2.2) pg/ml, respectively. CRP concentrations were higher in black compared to white patients (*P *= 0.002 and 0.03 before and after adjustment for confounders (see Table 3)) and those of interleukin-6 were similar in both groups (*P *= 0.4 and 0.4 before and after adjustment for confounders).

**Table 3:** Associations of potential determinants of systemic inflammation with CRP and interleukin-6 concentrations in all participants

	C-reactive protein^a^				Interleukin-6^a^			
		
Potential determinant	Age and sex adjusted model		Multivariable adjusted model		Age and sex adjusted model		Multivariable adjusted model	
	**Partial R**	***P***	**Partial R**	***P***	**Partial R**	***P***	**Partial R**	***P***
Body mass index	**0.240**	**0.0003**	-0.005	0.9	**0.227**	**0.0002**	**0.148**	**0.02**
Waist circumference	**0.318**	**<0.0001**			**0.173**	**0.005**		
Waist/height	**0.302**	**<0.0001**	**0.188**	**0.005**	**0.182**	**0.004**	-0.001	1.0
Waist/hip^a^	**0.163**	**0.01**			0.054	0.39		
Number of cigarettes/day^a^	0.017	0.8	0.034	0.6	**0.128**	**0.04**	**0.130**	**0.04**
Alcohol use	0.065	0.3	0.048	0.5	**0.162**	**0.008**	0.098	0.1
ACE inhibitor use	0.051	0.4	0.056	0.4	**-0.144**	**0.019**	**-0.127**	**0.045**
Model			0.350				0.331	

Significant associations are shown in bold. ^a^Non-normally distributed variables that were logarithmically transformed. Except for alcohol use (only in participants without rheumatoid arthritis), none of the relationships differed in black Africans with versus those without rheumatoid arthritis (interaction *P *= 0.1 to 0.9). CRP, C-reactive protein.

### Carotid artery atherosclerosis in African black subjects with and without RA

The carotid artery atherosclerosis burden in African black subjects with and without RA is also shown in Table 2. In age and sex adjusted analysis, the cIMT was similar in both groups (*P *= 0.7) (Model 1 in Table 2). Further adjustment for anthropometric measures, leflunomide and chloroquine use and systemic inflammation did not alter these results (models 2 to 5 in Table 2).

### Factors associated with systemic inflammation in African black subjects with and without RA

Table 3 gives the significant associations between the potential determinants of systemic inflammation that were recorded in subjects with and without RA (characteristics shown in Table 1) and serum CRP and interleukin-6 concentrations. In age- and sex-adjusted analysis, each anthropometric measure was associated with CRP concentrations and overall and abdominal obesity measures as well as the number of cigarettes smoked, alcohol use and angiotensin converting enzyme inhibitor therapy were associated with interleukin-6 concentrations. In additional models in which these characteristics (except for waist circumference and waist-hip ratio that were omitted because of co-linearity) were entered together as independent variables, the waist-height ratio remained associated with serum CRP concentrations and the BMI, number of cigarettes smoked per day and angiotensin converting enzyme inhibitor therapy were independently associated with interleukin-6 concentrations. None of the relationships between potential determinants of systemic inflammation and serum CRP and interleukin-6 concentrations differed in black African subjects with compared to those without RA (interaction *P *= 0.1 to 0.9).

Table 4 shows the analyses of the associations between recorded clinical disease activity and disease severity (deformed joint count) measures and serum CRP and interleukin-6 concentrations in subjects with RA. Both in age and sex adjusted models as well as in models that included additional adjustment for potential confounders (Table 3), there were no significant relationships; the same was true when the respective associations reassessed in subgroups with no or mild disease activity (Table 5) and moderate and high disease activity (Table 7), respectively. In fact, in those with more marked disease activity, a borderline inverse relationship between the swollen joint count and interleukin-6 concentrations was noted. These results were also unexpected. Hence, to ensure that the lack of association between CRP concentrations and clinical disease activity and severity measures is specific to African black patients with RA in our setting, we also assessed the respective relationships in African whites with established RA that formed part of our previously reported investigation [52]. Indeed, in these patients, upon adjustment for demographic characteristics, the log swollen joint count (partial R = 0.536, *P *< 0.0001), log tender joint count (partial R = 0.429, *P *< 0.0001), log doctor disease activity (partial R = 0.628, *P *< 0.0001), patient disease activity (partial R = 0.464, *P *< 0.0001), log CDAI (partial R = 0.580, *P *< 0.0001), CDAI >2.7 (partial R = 0.481, *P *< 0.0001) and log deformed joint count (partial R = 0.254, *P *= 0.005) were each strongly associated with log CRP concentrations. Upon further adjustment for additional potential confounders (Table 3), these relationships remained equally strong (partial R = 0.531, 0.425, 0.614, 0.460, 0.516, 0.462 and 0.284 for log swollen and log tender joint count, log doctor and patient disease activity, log CDAI and CDAI >2.7 and log deformed joint count (*P *< 0.003 for each), respectively).

**Table 4:** Relationships of disease activity and severity with systemic inflammation in all 115 African RA patients

	C-reactive protein^a^				Interleukin-6^a^			
		
Disease activity variable	Age and sex adjusted model		Multivariable adjusted model^b^		Age and sex adjusted model		Multivariable adjusted model^b^	
	**Partial R**	***P***	**Partial R**	***P***	**Partial R**	***P***	**Partial R**	***P***
Swollen joints^a^	0.081	0.4	0.102	0.3	-0.136	0.2	-0.101	0.3
Tender joints^a^	0.005	1.0	0.009	0.9	-0.029	0.8	-0.012	0.9
Doctor disease activity^a^	0.061	0.5	0.057	0.6	0.077	0.4	0.126	0.2
Patient disease activity	-0.053	0.6	0.082	0.4	0.101	0.3	0.090	0.4
CDAI^a^	0.065	0.5	0.049	0.6	0.083	0.4	0.031	0.8
CDAI >2.7	0.069	0.5	0.088	0.4	0.129	0.2	0.163	0.1
Deformed joints^a^	0.040	0.7	0.103	0.3	-0.027	0.8	-0.016	0.9

^a^Non-normally distributed variables that were logarithmically transformed; ^b^additionally adjusted for potential confounders of body mass index, waist:height ratio and angiotensin converting enzyme inhibitors (see Table 3). CDAI, Clinical Disease Activity Index.

**Table 5:** Relationships of disease activity and severity with systemic inflammation in non- or mildly active RA.

	C-reactive protein^a^				Interleukin-6^a^			
		
Disease activity variable	Age and sex adjusted model		Multivariable adjusted model^b^		Age and sex adjusted model		Multivariable adjusted model^b^	
	**Partial R**	***P***	**Partial R**	***P***	**Partial R**	***P***	**Partial R**	***P***
Swollen joints^a^	0.082	0.6	0.063	0.7	-0.189	0.2	-0.147	0.3
Tender joints^a^	-0.067	0.6	-0.100	0.5	-0.173	0.2	-0.168	0.3
Doctor disease activity^a^	0.044	0.8	0.017	0.9	-0.126	0.4	-0.027	0.9
Patient disease activity	-0.148	0.3	-0.139	0.3	0.070	0.6	0.011	0.9
CDAI^a^	-0.034	0.8	-0.060	0.7	-0.143	0.3	-0.116	0.4
Deformed joints^a^	0.071	0.6	0.119	0.4	-0.081	0.6	0.013	0.9

Fifty-four patients had no or mild disease activity (CDAI = 0 to 10). ^a^Non-normally distributed variables that were logarithmically transformed; ^b^additionally adjusted for potential confounders of body mass index, waist:height ratio and angiotensin converting enzyme inhibitors (see Table 3). CDAI, Clinical Disease Activity Index.

**Table 6:** Relationships of disease activity and severity with systemic inflammation in moderately or highly active RA

	C-reactive protein^a^				Interleukin-6^a^			
		
Disease activity variable	Age and sex adjusted model		Multivariable adjusted model^b^		Age and sex adjusted model		Multivariable adjusted model^b^	
	**Partial R**	***P***	**Partial R**	***P***	**Partial R**	***P***	**Partial R**	***P***
Swollen joints^a^	0.025	0.9	0.113	0.4	-0.260	0.07	-0.246	0.1
Tender joints^a^	0.028	0.8	0.014	0.9	-0.131	0.4	-0.120	0.4
Doctor disease activity^a^	0.100	0.5	0.084	0.6	0.212	0.2	0.232	0.1
Patient disease activity	-0.092	0.5	-0.066	0.7	0.222	0.1	0.225	0.2
CDAI^a^	0.066	0.6	0.111	0.5	0.125	0.4	0.147	0.3
Deformed joints^a^	0.041	0.8	0.028	0.8	0.012	0.9	-0.050	0.7

Sixty-one patients had moderate or high disease activity (CDAI > 10). ^a^Non-normally distributed variables that were logarithmically transformed; ^b^additionally adjusted for potential confounders of body mass index, waist:height ratio and angiotensin converting enzyme inhibitors (see Table 3). CDAI, Clinical Disease Activity Index.

Table 7 gives the significant associations of serum CRP and interleukin-6 concentrations with the recorded cardiovascular risk factors (Table 2) in African black subjects with and without RA. In age and sex adjusted models, both inflammatory markers were related to metabolic risk that comprised lipid variables. None of the associations between systemic inflammation and cardiovascular risk factors differed in African black subjects with compared to those without RA (interaction *P *= 0.4 to 0.9).

**Table 7:** Associations of systemic inflammation with cardiovascular risk factors in RA and non-RA African subjects

Cardiovascular risk factor	C-reactive protein^a^		Interleukin-6^a^	
		
	Partial R	*P*	Partial R	*P*
HDL chol^a^	**-0.247**	**0.0002**	-0.109	0.08
Chol/HDL chol	**0.208**	**0.002**	0.104	0.10
Triglycerides/HDL chol^a^	**0.170**	**0.01**	**0.147**	**0.02**
LDL chol/HDL chol^a^	**0.202**	**0.002**	0.038	0.5

Relationships were assessed in age and sex adjusted models. Significant associations are shown in bold. HDL, high density lipoprotein; chol, cholesterol; LDL, low density lipoprotein. None of the relationships differed in black Africans versus those without rheumatoid arthritis (interaction *P *= 0.4 to 0.9).

### Relationships between cardiovascular risk factors and carotid artery atherosclerosis in African subjects with and without RA

In age-, sex- and statin therapy-adjusted models, serum low-density lipoprotein (LDL) cholesterol concentrations were associated with cIMT (partial R = 0.153, *P *= 0.032). Further adjustment for other traditional risk factors comprising hypertension, diabetes and smoking did not materially alter these associations (R = 0.135, *P *= 0.06). The other recorded cardiovascular risk factors (Table 2) were not associated with cIMT and the relationships between cardiovascular risk factors and cIMT were consistently similar in black African subjects with compared to those without RA (interaction *P *> 0.2).

## Discussion

Among African black subjects from a developing population, patients with RA experienced markedly reduced adiposity compared to their non-RA counterparts. RA was not independently associated with hypertension, dyslipidemia, smoking and diabetes and the overall major traditional risk factor burden was similar in RA compared to non-RA subjects. Serum CRP concentrations were not increased in RA and further unrelated to disease activity and severity, a finding that was specific to African black patients with RA. The carotid artery atherosclerosis extent did not differ by RA status. To our knowledge, this is the first study that evaluated the association of RA with cardiovascular risk factors and atherosclerosis in African black persons.

In the present study, non-RA subjects experienced an apparently large systemic inflammation burden with mean circulating CRP and interleukin-6 concentrations of 6.7 mg/l and 6.3 pg/ml, respectively. Of likely relevance in the present context, the pro-inflammatory cytokine interleukin-6 *IL-6*-174 G/G genotype was found 36.5 (95% CI = 8.8 to 159.1) times more frequently in African compared to white Americans [65]. The most striking finding in the current investigation was the absence of increased systemic inflammation in RA compared to non-RA subjects despite the presence of clinically active disease [63] in >80% of the patients. This is in sharp contrast to the RA associated six- to seven-fold increase in both CRP and interleukin-6 concentrations in a study on mostly white patients with RA, as previously reported by us [21]. In addition, there was an overall lack of impact of RA on the relationships of circulating interleukin-6 and CRP concentrations with their potential determinants and metabolic risk factors. The concentrations of circulating interleukin-6 that constitute the major determinant of hepatic CRP production [12,17] were higher in non-RA compared to RA black Africans even after adjusting for confounders. However, the full impact of disparities in potential confounders including adiposity measures, smoking, alcohol consumption and angiotensin converting enzyme inhibitor use [16,66-68] on the association of RA status with circulating interleukin-6 concentrations may not have been accounted for in multivariable models, particularly given the cross-sectional design of our study.

Our findings have important implications. First, they suggest that interleukin-6 produced in inflamed joints is not released into the circulation in black Africans with established RA. As well, the very low prevalence of extra-articular manifestations among black Africans with RA [52,69] supports the presence of an inflammatory process that is mostly restricted to the joints. Since race specific therapeutic responses to interleukin-6 blockade with tocilizumab were not observed in randomized controlled trials that included black patients, interleukin-6 should be equally important in the pathogenesis of RA induced synovitis in black compared to other subjects [70].

Second, based on reported consistent results that originate in patients with RA from developed populations, a lack of adverse impact of RA on systemic inflammation would be expected to translate in unaltered cardiovascular risk factor profiles and disease [20,21,32,33,71,72]. Indeed, among African black subjects, RA was not related to adverse metabolic risk and atherosclerosis. Furthermore, whereas in patients with RA from developed populations, the associations of traditional cardiovascular risk factors with CVD are weakened due to the substantial contribution of systemic inflammation to cardiovascular mortality [31], in the present study, black Africans with RA experienced similar cardiovascular risk factor-atherosclerosis associations compared to their non-RA counterparts.

Third, our findings could explain the apparent disparities in RA-adiposity relationships among our patients and those that participated in previously reported studies [35-37,73,74]. Thus, patients with RA from developed populations sustain reduced lean body mass and increased adiposity that is mediated mainly by systemic inflammation and results in an overall unaltered or increased BMI and waist circumference, together with a more central fat distribution that enhances cardiovascular metabolic risk [35-37,73,74]. Accordingly, this condition has also been termed 'rheumatoid cachectic obesity' and 'hypercytokinaemic cachexia' [35]. In the present study, RA in black persons was associated with reduced overall and abdominal obesity indices and not with an altered fat distribution as estimated by waist-hip ratio. Waist circumference is determined by abdominal fat and hip circumference by lean mass and subcutaneous fat [75,76]. The waist and hip circumference were reduced to a similar extent in RA compared to non-RA subjects in this study. Reduced adiposity indices partially explained more favorable lipid profiles in RA in mixed regression models and, hence, may protect against CVD. Although systemic inflammation was not increased in African black patients with RA, their cumulative joint inflammation was distinctively large with a mean joint deformity count of 9.7. The joint deformity count is inversely related to BMI, waist and waist-height but not waist-hip ratio in African black women with RA [38] and the same relationships were found in the present investigation (data not shown). Reported findings together with our results therefore indicate that high-grade systemic and joint restricted inflammation may have disparate effects on adiposity and its distribution in RA.

Last, C-reactive protein concentrations should not be relied upon when determining the need for DMARD intensification in African black patients with RA.

Chloroquine, a long standing treatment in rheumatic diseases, can reduce hepatic cholesterol synthesis and increase LDL receptor numbers on fibroblasts and enhances insulin secretion and sensitivity [43-45]. We found that the associations of RA with a range of serum lipid concentrations and their ratios, including the triglycerides-HDL cholesterol ratio that is a marker of insulin resistance [77], were attenuated and no longer significant once chloroquine use was accounted for. Studies aimed at determining the true independent impact of RA on cardiovascular risk factor profiles should consider confounding by RA treatment.

Despite a low smoking prevalence and the small number of cigarettes consumed daily, the 10-year risk for CVD was substantial at approximately 10% in black Africans. These data are reminiscent of an earlier health transition stage that characterizes developing populations [49,50].

As previously reported in developed populations, RA was not associated with an augmented prevalence of hypertension in the present investigation [34]. Further, high frequencies of untreated and uncontrolled hypertension were documented earlier in RA [46] and, in black Africans, these also did not differ by RA status. Notably, however, despite similar blood pressure values to those in non-RA subjects, patients with RA employed more frequent and twice as many antihypertensive agents, a disparity that remained unexplained in multivariable analysis. Whether and how RA could influence hypertension responsiveness to antihypertensive therapy deserves to be determined in longitudinal studies.

The present study has further limitations. Carotid artery plaques are more strongly associated with coronary heart disease and its risk factors than cIMT that relates more closely to stroke and its determinants [38]. Nevertheless, both cIMT and plaque predict future cardiovascular event rates in RA and non-RA subjects irrespective of ethnicity [78-81]. Serum LDL concentrations were independently associated with cIMT that therefore would be expected to reflect atherosclerosis and coronary heart disease risk in black Africans. As applies to most studies on CVD, many relationships were evaluated. Our main findings each originated in confounder-adjusted multivariable models.

## Conclusions

RA associates with markedly reduced overall and abdominal adiposity in black Africans. However, in confounder adjusted analysis, RA did not impact on major traditional cardiovascular risk factor profiles, atherosclerosis extent and their relationships in this population. An absence of interleukin-6 release by inflamed RA joints into the circulation may account for this unaltered cardiovascular risk.

## Abbreviations

BMI: body mass index; BP: blood pressure; CDAI: Clinical Disease Activity Index; CHD: coronary heart disease; CI: confidence interval; cIMT: carotid intima-media thickness; CRP: C-reactive protein; CVD: cardiovascular disease; DMARDs: disease modifying drugs for rheumatic disease; HDL: high density lipoprotein; LDL: low density lipoprotein; NSAID: nonsteroidal anti-inflammatory drug; OR: odds ratio; RA: rheumatoid arthritis; RF: rheumatoid factor; SD: standard deviation.

## Competing interests

The authors declare that they have no competing interests.

## Authors' contributions

PHD contributed to the conception and design, performed the statistical analysis and drafted the manuscript. AJW and GRN provided the data in non-RA subjects and contributed to the conception and design, and analysis and interpretation of the data. AS provided the data in RA subjects in whom he also performed the ultrasound examinations and contributed to the conception and design, and revising the manuscript. All authors read and approved the final manuscript.

## References

[B1] van Sijl AM Peters MJ Knol DK de Vet HC Gonzalez-Gay MA Smulders YM Dijkmans BA Nurmohamed MT Carotid intima media thickness in rheumatoid arthritis as compared to control subjects: a meta-analysis Semin Arthritis Rheum 2011 15 389 397 10.1016/j.semarthrit.2010.06.006 20889191

[B2] Stamatelopoulos KS Kitas GD Papamichael CM Chryssohoou E Kyrkou K Georgiopoulos G Protogerou A Panoulas VF Sandoo A Tentolouris N Mavrikakis M Sfikakis PP Atherosclerosis in rheumatoid arthritis versus diabetes: a comparative study Arterioscler Thromb Vasc Biol 2009 15 1702 1708 10.1161/ATVBAHA.109.190108 19608975

[B3] Protogerou A Zampeli E Tentolouris N Makrilakis K Kitas G Sfikakis PP Subclinical femoral atheromatosis in rheumatoid arthritis: comparable prevalence to diabetes mellitus in a case-control study Ann Rheum Dis 2012 15 1534 1536 10.1136/annrheumdis-2011-201278 22764043

[B4] Kaplan MJ Cardiovascular complications of rheumatoid arthritis: assessment, prevention, and treatment Rheum Dis Clin North Am 2010 15 402 426 10.1016/j.rdc.2010.02.002 PMC2892739 20510241

[B5] Avina-Zubieta JA Thomas J Sadatsafavi M Lehman AJ Lacaille Risk of incident cardiovascular events in patients with rheumatoid arthritis: a meta-analysis of observational studies Ann Rheum Dis 2012 15 1524 1529 10.1136/annrheumdis-2011-200726 22425941

[B6] Peters MJ van Halm VP Voskuyl AE Smulders YM Boers M Lems WF Visser M Stehouwer CD Dekker JM Nijpels G Heine R Dijkmans BA Nurmohamed MT Does rheumatoid arthritis equal diabetes mellitus as an independent risk factor for cardiovascular disease? A prospective study Arthritis Rheum 2009 15 1571 1579 10.1002/art.24836 19877093

[B7] Avina-Zubieta JA Choi HK Sadatsafavi M Etminan M Esdaile JM Lacaille D Risk of cardiovascular mortality in patients with rheumatoid arthritis: a meta-analysis of observational studies Arthritis Rheum 2008 15 1690 1697 10.1002/art.24092 19035419

[B8] Expert Panel on Detection, Evaluation, and Treatment of High Blood Cholesterol in Adults Executive summary of the third report of the National Cholesterol Education Program (NCEP) Expert Panel on Detection, Evaluation, and Treatment of High Blood Cholesterol in Adults (Adult Treatment Panel III) JAMA 2001 15 2486 2497 10.1001/jama.285.19.2486 11368702

[B9] D'Agostino RB Sr Vasan RS Pencina MJ Wolf PA Cobain M Massaro JM Kannel WB General cardiovascular risk profile for use in primary care: the Framingham Heart Study Circulation 2008 15 743 753 10.1161/CIRCULATIONAHA.107.699579 18212285

[B10] Romano M Sironi M Toniatti C Polentanutti N Fruscella P Ghezzi P Faggioni R Luini W van Hinsbergh V Sozzani S Bussolino F Poli V Ciliberto G Mantovani A Role of IL-6 and its soluble receptor in induction of chemokines and leukocyte recruitment Immunity 1997 15 315 325 10.1016/S1074-7613(00)80334-9 9075932

[B11] Yudkin JS Stehouwer CDA Emeis JJ Coppack SW C-reactive protein in healthy subjects: associations with obesity, insulin resistance, and endothelial dysfunction. A potential role for cytokines originating from adipose tissue? Arterioscler Thromb Vasc Biol 1999 15 972 978 10.1161/01.ATV.19.4.972 10195925

[B12] Ridker PM Rifai N Stempfer MJ Hennekens CH Plasma concentration of interleukin-6 and the risk of future myocardial infarction among apparently healthy men Circulation 2000 15 1767 1772 10.1161/01.CIR.101.15.1767 10769275

[B13] Verma S Li S-H Badiwala MV Weisel RD Fedak PW Li R-K Dhillon B Mickle DA Endothelin antagonism and interleukin-6 inhibition attenuate the proatherogenic effects of C-reactive protein Circulation 2002 15 1890 1896 10.1161/01.CIR.0000015126.83143.B4 11997273

[B14] Ridker PM Buring JE Cook NR Rifai N C-reactive protein, the metabolic syndrome, and risk of incident cardiovascular events. An 8-year follow-up of 14 719 initially healthy American women Circulation 2003 15 391 397 10.1161/01.CIR.0000055014.62083.05 12551861

[B15] Rattazzi M Puato M Faggin E Bertipaglia B Zambon A Pauletto P C-reactive protein and interleukin-6 in vascular disease: culprits or passive bystanders? J Hypertens 2003 15 1787 1803 10.1097/00004872-200310000-00002 14508181

[B16] Pearson TA Mensah GA Alexander RW Anderson JL Cannon RO III Criqui M Fadl YY Fortmann SP Hong Y Myers GL Rifai N Smith SC Jr Taubert K Tracy RP Vinicor F Centers for Disease Control and Prevention; American Heart Association Markers of inflammation and cardiovascular disease: application to clinical and public health practice: a statement for healthcare professionals from the Centers for Disease Control and Prevention and the American Heart Association Circulation 2003 15 499 511 10.1161/01.CIR.0000052939.59093.45 12551878

[B17] Pepys MB Hirschfield GM C-reactive protein: a critical update J Clin Invest 2003 15 1805 1812 12813013 10.1172/JCI18921 PMC161431

[B18] Hansson GK Inflammation, atherosclerosis, and coronary artery disease N Engl J Med 2005 15 1685 1695 10.1056/NEJMra043430 15843671

[B19] Ridker PM C-reactive protein and the prediction of cardiovascular events among those at intermediate risk: moving an inflammatory hypothesis toward consensus J Am Coll Cardiol 2007 15 2129 2138 10.1016/j.jacc.2007.02.052 17531663

[B20] Sattar N McCarey DW Capell H McInnes IB Explaining how "high-grade" systemic inflammation accelerates vascular risk in rheumatoid arthritis Circulation 2003 15 2957 2963 10.1161/01.CIR.0000099844.31524.05 14676136

[B21] Dessein PH Joffe BI Singh S Biomarkers of endothelial dysfunction, cardiovascular risk factors and atherosclerosis in rheumatoid arthritis Arthritis Res Ther 2005 15 R634 R643 10.1186/ar1717 15899050 PMC1174955

[B22] Dessein PH Norton GR Woodiwiss AJ Joffe BI Solomon A Independent role of conventional cardiovascular risk factors as predictors of C-reactive protein concentrations in rheumatoid arthritis J Rheumatol 2007 15 681 688 17299845

[B23] Rodriguez-Rodriguez L Gonzalez-Juanatey C Palomino-Morales R Vazquez-Rodriguez TR Miranda-Filloy JA Fernandez-Gutierrez B Llorca J Martin J Gonzalez-Gay MA TNFA -308 (rs1800629) polymorphism is associated with a higher risk of cardiovascular disease in patients with rheumatoid arthritis Atherosclerosis 2011 15 125 130 10.1016/j.atherosclerosis.2010.10.052 21420089

[B24] Rodriguez-Rodriguez L Gonzalez-Juanatey C Garcia-Bermudez M Vazquez-Rodriguez TR Miranda-Filloy JA Fernandez-Gutierrez B Llorca J Martin J Gonzalez-Gay MA CCR5Δ32 variant and cardiovascular disease in patients with rheumatoid arthritis: a cohort study Arthritis Res Ther 2011 15 R133 10.1186/ar3444 21846359 PMC3239375

[B25] Teruel M Martin JE Gonzalez-Juanatey C Lopez-Mejias R Miranda-Filloy JA Blanco R Balsa A Pascual-Salcedo D Rodriguez-Rodriguez L Fernandez-Gutierrez B Ortiz AM Gonzalez-Alvaro I Gomez-Vaquero C Bottini N Llorca J Gonzalez-Gay MA Martin J Association of acid phosphatase locus 1*C allele with the risk of cardiovascular events in rheumatoid arthritis patients Arthritis Res Ther 2011 15 R116 10.1186/ar3401 21767392 PMC3239354

[B26] Gonzalez-Gay MA Gonzalez-Juanatey C Lopez-Diaz MJ Pineiro A Garcia-Porrua C Miranda-Filloy JA Ollier WE Martin J Llorca J HLA-DRB1 and persistent chronic inflammation contribute to cardiovascular events and cardiovascular mortality in patients with rheumatoid arthritis Arthritis Rheum 2007 15 125 132 10.1002/art.22482 17266100

[B27] Toms TE Panoulas VF Smith JP Douglas KM Metsios GS Stavropoulos-Kalinoglou A Kitas GD Rheumatoid arthritis susceptibility genes associate with lipid levels in patients with rheumatoid arthritis Ann Rheum Dis 2011 15 1025 1032 10.1136/ard.2010.144634 21398331

[B28] Solomon DH Curhan GC Rimm EB Cannuscio CC Karlson EW Cardiovascular risk factors in women with and without rheumatoid arthritis Arthritis Rheum 2004 15 3444 3449 10.1002/art.20636 15529391

[B29] del Rincon I Williams K Stern MP Freeman GL O'Leary DH Escalante A Association between carotid atherosclerosis and markers of inflammation in rheumatoid arthritis patients and healthy subjects Arthritis Rheum 2003 15 1833 1840 10.1002/art.11078 12847676

[B30] Kitas GD Gabriel SE Cardiovascular disease in rheumatoid arthritis: state of the art and future perspectives Ann Rheum Dis 2011 15 8 14 10.1136/ard.2010.142133 21109513

[B31] Gabriel SE Heart disease and rheumatoid arthritis: understanding the risks Ann Rheum Dis 2010 15 (Suppl 1):61 64 10.1136/ard.2009.119404 PMC4308040 19995747

[B32] Dessein PH Stanwix AE Joffe BI Cardiovascular risk in rheumatoid arthritis versus osteoarthritis: acute phase response related decreased insulin sensitivity and high-density lipoprotein cholesterol as well as clustering of metabolic syndrome features in rheumatoid arthritis Arthritis Res 2002 15 R5 10.1186/ar428 12223108 PMC125299

[B33] Dessein PH Joffe BI Insulin resistance and impaired beta cell function in rheumatoid arthritis Arthritis Rheum 2006 15 2765 2775 10.1002/art.22053 16947779

[B34] Boyer JF Gourraud PA Cantagrel A Davignon JL Constantin A Traditional cardiovascular risk factors in rheumatoid arthritis: A meta-analysis Joint Bone Spine 2011 15 179 183 10.1016/j.jbspin.2010.07.016 20851020

[B35] Rall LC Roubenoff R Rheumatoid cachexia: metabolic abnormalities, mechanisms and interventions Rheumatology 2004 15 1219 1223 10.1093/rheumatology/keh321 15292530

[B36] Summers GD Metsios GS Stavropoulos-Kalinoglou A Kitas GD Rheumatoid cachexia and cardiovascular disease Nat Rev Rheumatol 2010 15 445 451 10.1038/nrrheum.2010.105 20647995

[B37] Giles JT Allison M Blumenthal RS Post W Petri M Tracy R Szklo M Bathon JM Abdominal adiposity in rheumatoid arthritis: association with cardiometabolic risk factors and disease characteristics Arthritis Rheum 2010 15 3173 3182 10.1002/art.27629 20589684 PMC2962724

[B38] Solomon A Norton GR Woodiwiss AJ Dessein PH Obesity and carotid atherosclerosis in African black and Caucasian women with established rheumatoid arthritis: a cross-sectional study Arthritis Res Ther 2012 15 R67 10.1186/ar3784 22430029 PMC3446436

[B39] Dessein PH Joffe BI Suppression of circulating interleukin-6 concentrations is associated with decreased endothelial activation in rheumatoid arthritis Clin Exp Rheumatol 2006 15 161 167 16762151

[B40] Dessein PH Joffe BI Stanwix AE Christian BF Veller M Glucocorticoids and insulin sensitivity in rheumatoid arthritis J Rheumatol 2004 15 867 874 15124244

[B41] del Rincon I O'Leary DH Haas RW Escalante A Effect of glucocorticoids on the arteries in rheumatoid arthritis Arthritis Rheum 2004 15 3813 3822 10.1002/art.20661 15593231

[B42] Ruyssen-Witrand A Fautrel B Saraux A Le Loet X Pham T Cardiovascular risk induced by low-dose corticosteroids in rheumatoid arthritis: a systematic literature review Joint Bone Spine 2011 15 23 30 10.1016/j.jbspin.2010.02.040 20471897

[B43] Munro R Morrison E McDonald AG Hunter JA Madhok R Capell HA Effect of disease modifying agents on the lipid profiles of patients with rheumatoid arthritis Ann Rheum Dis 1997 15 374 377 10.1136/ard.56.6.374 9227167 PMC1752398

[B44] Dessein PH Christian BF Solomon A Which are the determinants of dyslipidemia in rheumatoid arthritis and does socioeconomic status matter in this context? J Rheumatol 2009 15 1357 1361 10.3899/jrheum.090288 19567631

[B45] Wasko MC Hubert HB Lingala VB Elliott JR Luggen ME Fries JF Ward MM Hydroxychloroquine and risk of diabetes in patients with rheumatoid arthritis JAMA 2007 15 187 193 10.1001/jama.298.2.187 17622600

[B46] Panoulas VF Metsios GS Pace AV John H Treharne GJ Banks MJ Kitas GD Hypertension in rheumatoid arthritis Rheumatology 2008 15 1286 1298 10.1093/rheumatology/ken159 18467370

[B47] Laborde F Loeuille D Chary-Valckenaere I Life-threatening hypertriglyceridemia during leflunomide therapy in a patient with rheumatoid arthritis Arthritis Rheum 2004 15 3398 15476234 10.1002/art.20498

[B48] Yusuf S Hawken S Ounpuu S Dans T Avezum A Lanas F McQueen M Budaj A Pais P Varigos J Lisheng L INTERHEART Study Investigators Effect of potentially modifiable risk factors associated with myocardial infarction in 52 countries (the INTERHEART study): case-control study Lancet 2004 15 937 952 10.1016/S0140-6736(04)17018-9 15364185

[B49] Yusuf S Reddy S Ounpuu S Anand S Global burden of cardiovascular diseases: part 1: general considerations, the epidemiologic transition, risk factors, and impact of urbanization Circulation 2001 15 2746 2753 10.1161/hc4601.099487 11723030

[B50] Solomon A Christian BF Norton GR Woodiwiss AJ Dessein PH Risk factor profiles for atherosclerotic cardiovascular disease in black and other Africans with established rheumatoid arthritis J Rheumatol 2010 15 953 960 10.3899/jrheum.091032 20231201

[B51] Dessein PH Norton GR Joffe BI Abdool-Carrim AT Woodiwiss AJ Solomon A Metabolic cardiovascular risk burden and atherosclerosis in African black and Caucasian women with rheumatoid arthritis: a cross-sectional study Clin Exp Rheumatol 2013 15 53 61 22935383

[B52] Solomon A Woodiwiss AJ Abdool-Carrim AT Stevens BA Norton GR Dessein PH The carotid artery atherosclerosis burden and its relation to cardiovascular risk factors in black and white Africans with established rheumatoid arthritis: a cross-sectional study J Rheumatol 2012 15 1798 1806 10.3899/jrheum.120073 22753659

[B53] Puoane T Steyn K Bradshaw D Laubscher R Fourie J Lambert V Mbanaga N Obesity in South Africa: the South African Demographic and Health Survey Obes Res 2002 15 1038 1048 10.1038/oby.2002.141 12376585

[B54] Opie LH Seeadat YK Hypertension in Sub-Saharan African populations Circulation 2005 15 3562 3568 10.1161/CIRCULATIONAHA.105.539569 16330697

[B55] Schutte AE Schutte R Huisman HW van Rooyen JM Fourie CM Malan NT Malan L Mels CM Smith W Moss SJ Towers GW Kruger HS Wentzel-Viljoen E Vorster HH Kruger A Are behavioural risk factors to be blamed for the conversion from optimal blood pressure to hypertensive status in Black South Africans? A 5-year prospective study Int J Epidemiol 2012 15 1114 1123 10.1093/ije/dys106 22825590

[B56] Redelinghuys M Norton GR Janse van Rensburg NM Maseko MJ Majane OH Dessein P Woodiwiss AJ Lack of independent association between C-reactive protein and central aortic hemodynamics in black Africans with high risk of cardiovascular disease Am J Hypertens 2011 15 1094 1101 10.1038/ajh.2011.119 21753803

[B57] Schutte AE van Vuuren D van Rooyen JM Huisman HW Schutte R Malan L Malan NT Inflammation, obesity and cardiovascular function in African and Caucasian women from South Africa: the POWIRS study J Hum Hypertens 2006 15 850 859 10.1038/sj.jhh.1002065 16855625

[B58] Phulukdaree A Khan S Ramkaran P Govender R Moodley D Chuturgoon AA The interleukin-6 -147 g/c polymorphism is associated with increased risk of coronary artery disease in young South African Indian men Metab Syndr Relat Disord 2013 15 205 209 10.1089/met.2012.0130 23461479

[B59] Norton GR Maseko M Libhaber E Libhaber CD Majane OH Dessein P Sareli P Woodiwiss AJ Is prehypertension an independent predictor of target organ changes in young-to-middle-aged persons of African descent? J Hypertens 2008 15 2279 2287 10.1097/HJH.0b013e328311f296 19008706

[B60] Woodiwiss AJ Molebatsi N Maseko MJ Libhaber E Libhaber C Majane OH Paiker J Dessein P Brooksbank R Sareli P Norton GR Nurse-recorded auscultatory blood pressure at a single visit predicts target organ changes as well as ambulatory blood pressure J Hypertens 2009 15 287 297 10.1097/HJH.0b013e328317a78f 19155786

[B61] Norton GR Majane OH Maseko MJ Libhaber C Redelinghuys M Kruger D Veller M Sareli P Woodiwiss AJ Brachial blood pressure-independent relations between radial late systolic shoulder-derived aortic pressures and target organ changes Hypertension 2012 15 885 892 10.1161/HYPERTENSIONAHA.111.187062 22331378

[B62] Arnett FC Edworthy SM Bloch DA McShane DJ Fries JF Cooper NS Healey LA Kaplan SR Liang MH Luthra HS Medsger TA Mitchell DM Neustadt DH Pinals RS Schaller JG Sharp JT Wilder RL Hunder GG The American Rheumatism Association 1987 revised criteria for the classification of rheumatoid arthritis Arthritis Rheum 1988 15 315 324 10.1002/art.1780310302 3358796

[B63] Gulfe A Aletaha D Saxne T Geborek P Disease activity level, remission and response in established rheumatoid arthritis: performance of various criteria sets in an observational cohort, treated with anti-TNF agents BMC Musculoskelet Disord 2009 15 41 10.1186/1471-2474-10-41 19389230 PMC2678269

[B64] Gepner AD Korcarz CE Aeschlimann SE LeCaire TJ Palta M Tzou WS Stein JH Validation of a carotid intima-media thickness border detection program for use in an office setting J Am Soc Echocardiogr 2006 15 223 228 10.1016/j.echo.2005.09.006 16455429

[B65] Ness RB Haggerty CL Harger G Ferrell R Differential distribution of allelic variants in cytokine genes among African Americans and white Americans Am J Epidemiol 2004 15 1033 1038 10.1093/aje/kwh325 15561982

[B66] Khoruts A Stahnke L McClain CJ Logan G Allen JI Circulating tumor necrosis factor, interleukin-1 and interleukin-6 concentrations in chronic alcoholic patients Hepatology 1991 15 267 276 1995437

[B67] Di Napoli M Papa F Angiotensin-converting enzyme inhibitor use is associated with reduced plasma concentration of C-reactive protein in patients with first-ever ischemic stroke Stroke 2003 15 2922 2929 10.1161/01.STR.0000099124.84425.BB 14605324

[B68] Tsutamoto T Wada A Maeda K Mabuchi N Hayashi M Tsutsui T Ohnishi M Sawaki M Fujii M Matsumoto T Kinoshita M Angiotensin II type 1 receptor antagonist decreases plasma levels of tumor necrosis factor alpha, interleukin-6 and soluble adhesion molecules in patients with chronic heart failure J Am Coll Cardiol 2000 15 714 721 10.1016/S0735-1097(99)00594-X 10716475

[B69] Chikanza IC Stein M Lutalo S Gibson T The clinical, serologic and radiologic features of rheumatoid arthritis in ethnic black Zimbabwean and British Caucasian patients J Rheumatol 1994 15 2011 2015 7869302

[B70] Genovese MC McKay JD Nasonov EL Mysler EF da Silva NA Alecock E Woodworth T Gomez-Reino JJ Interleukin-6 receptor inhibition with tocilizumab reduces disease activity in rheumatoid arthritis with inadequate response to disease-modifying antirheumatic drugs Arthritis Rheum 2008 15 2968 2980 10.1002/art.23940 18821691

[B71] Dessein PH Tobias M Veller MG Metabolic syndrome and subclinical atherosclerosis in rheumatoid arthritis J Rheumatol 2006 15 2425 2432 17080519

[B72] Chung CP Oeser A Solus JF Avalos I Gebretsadik T Shintani A Raggi P Sokka T Pincus T Stein CM Prevalence of the metabolic syndrome is increased in rheumatoid arthritis and is associated with coronary atherosclerosis Atherosclerosis 2008 15 758 763 10.1016/j.atherosclerosis.2007.01.004 17266963

[B73] Westhovens R Nijs J Taelman V Dequeker J Body composition in rheumatoid arthritis Br J Rheumatol 1997 15 444 448 10.1093/rheumatology/36.4.444 9159537

[B74] Giles JT Ling SM Ferrucci L Bartlett SJ Andersen RE Towns M Muller D Fontaine KR Bathon JM Abnormal body composition phenotypes in older rheumatoid arthritis patients: association with disease characteristics and pharmacotherapies Arthritis Rheum 2008 15 807 815 10.1002/art.23719 18512711 PMC2670994

[B75] Hassinen M Lakka TA Komulainen P Haapala I Nissinen A Rauramaa R Association of waist and hip circumference with 12-year progression of carotid intima-media thickness in elderly women Int J Obes 2007 15 1406 1411 10.1038/sj.ijo.0803613 17372615

[B76] Snijder MB Zimmet PZ Visser M Dekker JM Seidell JC Shaw JE Independent association of hip circumference with metabolic profile in different ethnic groups Obes Res 2004 15 1370 1374 10.1038/oby.2004.173 15483201

[B77] Dessein PH Joffe BI Stanwix AE Subclinical hypothyroidism is associated with insulin resistance in rheumatoid arthritis Thyroid 2004 15 443 446 15242571 10.1089/105072504323150750

[B78] Gonzalez-Juanatey C Llorca J Martin J Gonzalez-Gay MA Carotid intima-media thickness predicts the development of cardiovascular events in patients with rheumatoid arthritis Semin Arthritis Rheum 2009 15 366 371 10.1016/j.semarthrit.2008.01.012 18336869

[B79] Evans MR Escalante A Battafarano DF Freeman GL O'Leary DH del Rincon I Carotid atherosclerosis predicts incident acute coronary syndrome in rheumatoid arthritis Arthritis Rheum 2011 15 1211 1220 10.1002/art.30265 21305526 PMC3286362

[B80] Greenland P Alpert JS Beller GA Benjamin EJ Budoff MJ Fayad ZA Foster E Hlatky MA Hodgson JM Kushner FG Lauer MS Shaw LJ Smith SC Jr Taylor AJ Weintraub WS Wenger NK Jacobs AK Smith SC Jr Anderson JL Albert N Buller CE Creager MA Ettinger SM Guyton RA Halperin JL Hochman JS Kushner FG Nishimura R Ohman EM Page RL 2010 ACCF/AHA guideline for assessment of cardiovascular risk in asymptomatic adults: a report of the American College of Cardiology Foundation/American Heart Association Task Force on practice guidelines J Am Coll Cardiol 2010 15 e50 103 10.1016/j.jacc.2010.09.001 21144964

[B81] Peters SA den Ruijter HM Bots ML Moons KG Improvements in risk stratification for the occurrence of cardiovascular disease by imaging subclinical atherosclerosis: a systematic review Heart 2012 15 177 184 10.1136/heartjnl-2011-300747 22095617

